# Associations of Insulin and Insulin-Like Growth Factors with Physical Performance in Old Age in the Boyd Orr and Caerphilly Studies

**DOI:** 10.1371/journal.pone.0030096

**Published:** 2012-01-10

**Authors:** Kate Birnie, Yoav Ben-Shlomo, Jeff M. P. Holly, David Gunnell, Shah Ebrahim, Antony Bayer, John Gallacher, Richard M. Martin

**Affiliations:** 1 School of Social and Community Medicine, University of Bristol, Bristol, United Kingdom; 2 School of Clinical Sciences, University of Bristol, Bristol, United Kingdom; 3 London School of Hygiene & Tropical Medicine, London, United Kingdom; 4 School of Medicine, Cardiff University, Cardiff, United Kingdom; Brigham & Women's Hospital, and Harvard Medical School, United States of America

## Abstract

**Objective:**

Insulin and the insulin-like growth factor (IGF) system regulate growth and are involved in determining muscle mass, strength and body composition. We hypothesised that IGF-I and IGF-II are associated with improved, and insulin with worse, physical performance in old age.

**Methods:**

Physical performance was measured using the get-up and go timed walk and flamingo balance test at 63–86 years. We examined prospective associations of insulin, IGF-I, IGF-II and IGFBP-3 with physical performance in the UK-based Caerphilly Prospective Study (CaPS; n = 739 men); and cross-sectional insulin, IGF-I, IGF-II, IGFBP-2 and IGFBP-3 in the Boyd Orr cohort (n = 182 men, 223 women).

**Results:**

In confounder-adjusted models, there was some evidence in CaPS that a standard deviation (SD) increase in IGF-I was associated with 1.5% faster get-up and go test times (95% CI: −0.2%, 3.2%; p = 0.08), but little association with poor balance, 19 years later. Coefficients in Boyd Orr were in the same direction as CaPS, but consistent with chance. Higher levels of insulin were weakly associated with worse physical performance (CaPS and Boyd Orr combined: get-up and go time = 1.3% slower per SD log-transformed insulin; 95% CI: 0.0%, 2.7%; p = 0.07; OR poor balance 1.13; 95% CI; 0.98, 1.29; p = 0.08), although associations were attenuated after controlling for body mass index (BMI) and co-morbidities. In Boyd Orr, a one SD increase in IGFBP-2 was associated with 2.6% slower get-up and go times (95% CI: 0.4%, 4.8% slower; p = 0.02), but this was only seen when controlling for BMI and co-morbidities. There was no consistent evidence of associations of IGF-II, or IGFBP-3 with physical performance.

**Conclusions:**

There was some evidence that high IGF-I and low insulin levels in middle-age were associated with improved physical performance in old age, but estimates were imprecise. Larger cohorts are required to confirm or refute the findings.

## Introduction

Increasing life expectancy in the UK has provoked public health concern about the prospects of a growing number of people experiencing functional limitations in old age. Understanding the causes of poor physical functioning and the identification of potential prevention strategies are critically important public health issues. One possible pathway contributing to poor physical functioning is the insulin-like growth factor (IGF) system. The IGF family contains two peptide hormones, or growth factors: IGF-I and IGF-II; and six binding proteins IGFBP-1 to -6 that modulate IGF activity [1]. Ageing is associated with a decline in levels of IGF-I, which may reflect decreases in growth hormone production with age [2]. The IGF system is involved in body composition, muscle maintenance and bone cell survival [3] and might influence measures of physical function in old age via a loss of muscle mass and strength [4]. A previous cross-sectional study indicates that high levels of IGF-I are weakly associated with faster walking speed [5] but results have not always been consistent [6], [7]. Other components of the IGF system may also be associated with functional ability with age: high IGF-I increases sensitivity to insulin [8] which may in turn be related to increased muscle strength [9]; circulating levels of IGFBP-2 are reduced in people with obesity [10] and insulin resistance [7], [11], [12] and may mediate associations of obesity and insulin resistance with poor physical functioning; higher circulating IGFBP-3 lowers the bioavailability of IGF-I, so may be associated with poorer physical performance, although, IGFBPs are thought to exert both positive and negative regulatory effects on IGF activity [13]. The role of IGF-II is less well known, although it has been suggested to play a critical role in muscle regeneration, and relatively higher IGF-II levels may prevent the age-related decline in muscle mass [14]. Understanding the role of the insulin-IGF system in physical performance has potential public health implications because it is nutritionally regulated, e.g. higher milk intake increases IGF-I [15], and IGFs are linked with other lifestyle factors, e.g. IGF-I and IGFBP-3 have been positively associated with increased physical activity and reduced cigarette smoking [16], so may be potentially modifiable.

Cross sectional studies cannot demonstrate the direction of association but prospective studies to investigate associations of the IGF system with physical performance have not been conducted to date. Here we examine prospective associations of the IGF system and physical performance 19 years later in the Caerphilly Prospective Study (CaPS) and compare these to cross-sectional associations of the IGF system and physical performance in old age in the Boyd Orr cohort. Our *a priori* hypothesis was that low levels of IGF-I and IGF-II and higher levels of insulin would be associated with reduced physical performance in old age.

## Materials and Methods

### Participants

The Boyd Orr study is an historical cohort based on the Carnegie (Boyd Orr) Survey of Diet and Health in Pre-War Britain, 1937–1939 [17]. In the original survey, 4,999 boys and girls aged 0–19 years in 16 centers across the UK underwent physical measurements, the family completed a week-long dietary inventory and detailed assessments of socioeconomic environment were made. Ethical approval for the Boyd Orr study was obtained from the Multicentre Research Ethics Committee for Scotland. In 1997, a follow-up study re-established contact with the subjects using the National Health Service Central Register and its equivalent in Edinburgh. The follow-up study identified 3,182 individuals from the original sample of families who were traced, alive and residing in Britain. These subjects were sent a detailed health and lifestyle questionnaire and 1,648 responses were received. In 2002, all 732 surviving study members aged 63–83 years who lived near clinics in Bristol, London, Wisbech, Aberdeen and Dundee, and had previously consented to clinical follow-up, were contacted; of these 405 (55%) - 182 men and 223 women - agreed to take part in a detailed clinical examination and questionnaire, where fasting blood samples were taken and the get-up and go walking speed [18] and flamingo balance [19] physical performance tests were carried out.

The Caerphilly Prospective Study (CaPS) recruited 2,512 men aged 45–59 years between 1979 and 1983 from the town of Caerphilly, South Wales and the adjacent villages (http://www.epi.bris.ac.uk/caerphilly/caerphillyprospectivestudy.htm) [20]. Ethical approval for the different phases of the study was given by the Ethics Committee of the Division of Medicine of the former South Glamorgan Area Health Authority and Gwent Research Ethics Committee. For the second examination (phase II 1984–1988), the original cohort was supplemented with 447 men of a similar age who had moved into the defined area; however, 561 men were lost from the cohort giving a total of 2,398 men who participated in phase II. Since then, the men have been examined on three further occasions: phases III (1989–1993), IV (1993–1996) and V (2002–2004). At each phase, the men completed lifestyle questionnaires and fasting blood samples were taken. A total of 1,195 men aged 66–86 years attended the phase V clinic (or had a home visit) where the physical performance tests were undertaken. All participants in both cohorts gave informed written consent.

### Measurement of physical performance

#### The get-up and go test

This is a standardized objective measure of functional leg strength, power, mobility and balance that is strongly correlated with activities of daily living and integrates a number of basic mobility movements considered necessary for successful ageing and protection from disability [18]. The participant is timed while they rise from a chair, walk three meters, turn, walk back to the chair and sit down. The test was performed on each person twice and the mean value used in the analysis to minimize measurement error.

#### The flamingo test

Measures the ability to maintain postural stability in the upright position, a pre-requisite for locomotor mobility [19]. The flamingo test was performed by timing how long subjects could lift one leg, whilst the other leg remained straight, with their eyes open. The position was held for as long as possible, for a maximum of 30 seconds. Participants used their preferred leg to stand on. The flamingo test was performed twice, unless the maximum score of 30 seconds was achieved in the first attempt. The best score was used in the analysis. The tests were measured using the same standardized protocol in both cohorts and the researchers trained by the same investigator (RMM).

### Measurement of insulin and insulin-like growth factors

In Boyd Orr, IGF-I and IGF-II, the major binding proteins (IGFBP-2 and IGFBP-3) and insulin were measured from blood samples taken from the 405 participants in the 2002 clinic. Samples were taken after an overnight fast. The samples were spun and frozen to −20°C within one hour and transferred within three weeks to a −80°C freezer [21]. In CaPS, fasting insulin was assayed for previous studies and was measured using a radioimmunoassay based on a double antibody for insulin, available for 1,005 men with physical performance data. Insulin was used from phase I if available, or if the men did not attend phase I, data from phase II was used (n = 186). Serum IGF-I, IGF-II and IGFBP-3 were measured on 739 samples stored at −80°C and selected from phase II, approximately 19 years prior to the physical performance tests. Serum IGF-I, IGF-II, and IGFBP-3 levels were measured using in-house double-antibody radio immunoassays in both cohorts [22]. The radioimmunoassay measures total IGF-I and IGF-II levels, including all forms that have undergone minor fragmentation. In Boyd Orr, total levels of IGFBP-2 were measured by one-step sandwich enzyme-linked immunosorbent assays (DSL-10-7100; Diagnostic Systems Laboratories, Webster, TX); insulin was measured with an insulin-specific assay that does not cross-react with proinsulin and split forms of proinsulin. In Boyd Orr, the average coefficients of variation for intraassay variability for IGF-I, IGF-II, IGFBP-3, and IGFBP-2 were 6.7, 10.0, 3.9, and 5.0% and for interassay variation were 9.7, 14.0, 8.1, and 7.1%. Interassay coefficients of variation were 5.3%, 4.2% and 4.4% for low, medium and high values of insulin. In CaPS, the average coefficients of variation for intraassay variability for IGF-I, IGF-II, IGFBP-3 and phase II insulin were 7.0, 7.9, 5.6 and 7.5% and for interassay variation were 8.8, 9.7, 8.8 and 8.9%.

### Measurement of potential confounders

Information on potential confounders was collected from questionnaires administered at research clinics; in Boyd Orr this was from 1997 and 2002, and in CaPS this was from phases I (1979–1983), II (1984–1988) and III (1989–1993) and has been described in detail previously [23]. In Boyd Orr, adult social class was recorded in 1997 and corresponded to the job participants spent most of their working lives. For men, adult social class was classified into categories based on the 1966 Classification of Occupations. For women, the social class of their spouse or partner was used, if available, otherwise their own occupation was used. Adult social class was classified into three categories in Boyd Orr (I/II, professional and managerial; III, skilled; IV/V, partly skilled, unskilled, other or unclassifiable). In CaPS, social class in adulthood was based on participants' present or last job at phase II (1984–1988) and divided into four categories (I/II, professional and managerial; III, skilled non-manual; III, skilled manual; IV/V, partly skilled, unskilled, other or unclassifiable). Health behaviors were recorded at the 2002 Boyd Orr clinic and CaPS phase II. Questions on smoking behavior were used to assign people into three groups (current; past; and never smokers). Alcohol consumption was coded into three groups (frequent [at least weekly]; occasional; and never). Exercise scores were derived from questions on the frequency physical activities were carried out. The overall scores were split into three approximately equal sized groups ranging from least to most exercise. Adult diet was estimated from food frequency questionnaires and averaged over multiple time periods to reduce measurement error (Boyd Orr 1997 and 2002; CaPS phases I to III). To calculate nutrient intake, frequency of consumption of a food was multiplied by the nutrient content of a standard portion of that food using food tables [24]. In CaPS, the average portion sizes used were calculated from seven-day weighed dietary records completed by a one-third sample of the Caerphilly cohort at phase I [25]. Milk consumption, calcium, protein, fat and energy intake have been linked to IGFs in many cohorts [15], [26]–[28], so these were considered as potential dietary confounding variables. Intake of milk was split into four groups ranging from lowest to highest intake; calcium, protein, fat and energy intake were considered as continuous variables.

### Measurement of body mass index and co-morbidities

Body mass index (BMI) was derived from height and weight measurements (weight [kg]/height [m]^2^), which were recorded at the research clinics when the blood samples were taken. We control for BMI because a previous study in the Boyd Orr cohort found a negative association between BMI and IGFBP-2, an inverted U-shape relationship between BMI and IGF-I, and a positive relationship of BMI with IGF-II and insulin [12]. Increased BMI was associated with slower walking times and poor balance in both cohorts [23]. Information on major co-morbidities (stroke, cancer, diabetes and angina) was collected from self-reported questionnaires. Derived variables were created to indicate a diagnosis of each disease by the time of the physical performance examination. In Boyd Orr: a diagnosis of diabetes was derived from a combination of self-report and fasting glucose measurement; cancer prevalence at the time of the measurement of physical performance was based on either self-reported cancer or a notification of cancer registration received from the National Health Service Central Register. Angina was based on the Rose chest pain questionnaire [29].

### Statistical analyses

All analyses were carried out using Stata version 11. IGF values and levels of insulin were converted into z-scores, which accounted for any baseline and laboratory related variation in IGF levels: age at blood sample; sex (Boyd Orr); duration of sample storage time; the laboratory technician who performed the assays (CaPS); and the week of the assay (CaPS). The z-scores have a mean of zero and a standard deviation (SD) of one, enabling the comparison for an equivalent one SD change in each IGF measurement. Due to skewed distributions, IGFBP-2 and insulin and were natural log transformed before the z-scores were created. The IGF-I∶BP-3 molar ratio may be a marker of IGF-I bioavailability. Based on the molecular weight of IGF-I (7500) and IGFBP-3 (40,000, mean of glycosylated variants), the molar ratio was calculated by multiplying IGF-I∶BP-3 by 5.33 (40,000∶7500) [21]. Blood samples for IGF measurements (CaPS phase II) were only available for 739 men with the physical performance outcomes (CaPS phase V). Any differences for those with and without stored blood samples were examined with chi-squared tests. Correlations between continuous variables were calculated using Pearson's correlation coefficients.

Walking times from the get-up and go test were natural log-transformed, due to a skewed distribution. Linear regression models were used to investigate associations of IGFs with the log-transformed walking time and regression coefficients were multiplied by 100 to represent percentage change in walk time [30]. Since a longer time to complete the get-up and go test implies person was slower, a *positive* coefficient of percentage change represents a *slower* walking time and a *negative* estimated coefficient of percentage change represents a *faster* walking time. Over a third of participants achieved the maximum balance of 30 seconds; therefore, the flamingo test was dichotomized at the lowest 20% of performers, using a cut-point of five seconds. The flamingo test was modeled using logistic regression, with the outcome of interest being the inability to balance for five seconds (coded with a value of one in models and termed ‘poor balance’ in the text). We tested for interactions between the cohorts to assess whether it was suitable to pool the data. Minimally adjusted models control for age at the time of the physical performance test and sex (Boyd Orr); further adjusted models additionally control for research centre, adult social class, diet, exercise, smoking status and alcohol consumption; final models also controlled for BMI and co-morbidities (stroke, cancer diabetes and angina) at the time of the IGF measurement. Non-linear associations for the IGF variables were investigated by comparing models with and without quadratic terms.

## Results

Descriptive statistics for the outcomes, exposures and potential confounding variables are shown in Table 1. In Boyd Orr, the physical performance tests and IGF measurements were carried out when the participants were a mean age of 70.7 years (inter-quartile range [IQR] 67–74; range 63–83 years). CaPS men were older at the time of the physical performance tests (mean age 75.3; IQR 71–78; range 66–86 years) and their blood samples for the IGF measurements were taken approximately 19 years earlier (mean age 56.1; IQR 52–60; range 47–67 years).

**Table 1:** Characteristics of study members.

	Mean (SD); median (quartiles)*; or N (%)			
	N	Boyd Orr	N	CaPS
**Outcomes**				
Get-up and go test (seconds)*	405	9.3 (8.2, 10.8)	1114	10.3 (9.0, 12.2)
Flamingo test (balance <5 seconds)	404	81 (20%)	1123	290 (26%)
**Exposures**				
IGF-I (ng/ml)	405	131.4 (40.9)	739	155.4 (48.1)
IGF-II (ng/ml)	405	746.6 (226.4)	739	719.1 (238.1)
IGFBP-2 (ng/ml)*	405	415.0 (280.0, 597.3)	-	
IGFBP-3 (ng/ml)	405	4138.2 (1008.7)	739	3387.8 (757.9)
Insulin (mu/l)*	370	7.8 (5.4, 12.5)	1005	5.3 (3.3, 9.0)
**Potential confounders**				
Age at clinic where blood sample was taken (years)	405	70.7 (4.3)	1195	56.1 (4.4)
Age at performance tests (years)	405	70.7 (4.3)	1195	75.3 (4.3)
Sex (male)	405	182 (45%)	1195	1195 (100%)
Adult social class	399		928	
I/II		28 (7%)		77 (8%)
III NM†		} 97 (24%)		55 (6%)
III M†				591 (64%)
IV/V/Other‡		192 (48%)		166 (18%)
Unemployed		82 (21%)		39 (4%)
Smoking status	405		1139	
Current		43 (11%)		407 (36%)
Past		198 (49%)		470 (41%)
Never		164 (40%)		262 (23%)
Alcohol consumption	404		1141	
At least weekly		196 (49%)		790 (69%)
Occasionally		152 (38%)		286 (25%)
Never		56 (14%)		65 (6%)
Exercise group	404		1141	
1 (Least)		130 (32%)		329 (29%)
2		121 (30%)		423 (37%
3 (Most)		153 (38%)		389 (34%)
Adult diet				
Calcium (mg)	405	1109 (373)	1190	926 (219)
Protein (g)	405	88 (25)	1193	72 (14)
Fat (g)	405	76 (30)	1192	87 (22)
Total energy (kcal)	402	2206 (697)	1190	2109 (452)
Milk group§	395		1193	
Milk group: none		11 (3%)		130 (11%)
Less than half a pint		101 (25%)		621 (52%)
Half a pint to a pint		196 (48%)		398 (33%)
More than a pint		97 (24%)		44 (4%)
BMI (kg/m^2^)	405	27.5 (4.4)	1178	26.6 (3.4)
Stroke	403	22 (5.5%)	1102	131 (11.9%)
Cancer	405	58 (14.3%)	1100	150 (13.6%)
Diabetes	402	39 (9.7%)	1096	158 (14.4%)
Angina	401	75 (18.7%)	1090	236 (21.7%)

Note: sample sizes vary due to missing data. Blood samples from CaPS phase II for IGFs were only available for 739 (62%) of the 1,195 men who attended the CaPS phase V clinic.

SD = Standard deviation.

*Median and quartiles are presented, due to skewed distributions; the mean (SD) for IGFBP-2 was: 484.2 (292.3) ng/ml and for insulin: 10.0 (6.9) mu/l in Boyd Orr; 7.6 (14.8) mu/l in CaPS.

† Data are not available to distinguish between the social classes III manual (M) and III non-manual (NM) in Boyd Orr.

‡ Armed forces, unemployed or unclassifiable.

§ Milk groups range from lowest to highest intake: none, ½ pint (∼237 g) or less, ½ pint (∼237 g) to one pint (∼473 g), more than one pint (∼473 g).

Blood samples from CaPS phase II were available for 739 (62%) of the 1,195 men who attended the CaPS phase V clinic. There were no important differences in potential confounders (e.g. age, smoking, social class, alcohol use, physical activity) for those with and without blood samples. IGF-I was positively correlated with IGF-II and IGFBP-3, but inversely correlated with IGFBP-2 (Table 2). IGF-II was positively correlated with IGFBP-3 and negatively correlated with IGFBP-2. The IGF binding proteins were negatively correlated with each other and IGFBP-2 was negatively correlated with insulin. Associations of social class and lifestyle factors have already been published for these cohorts, briefly, more deprived social class, cigarette smoking, lower levels of exercise, greater BMI, and a history of stroke and angina were all associated with worse physical performance.

**Table 2:** Pearson correlation coefficients of the insulin-like growth factor system in Boyd Orr and CaPS.

	Correlation coefficient (p value)			
	IGF-I	IGF-II	IGFBP-2*	IGFBP-3
**IGF-II**				
Boyd Orr	0.39 (<0.001)			
CaPS	0.27 (<0.001)			
**IGFBP-2** *				
Boyd Orr	−0.19 (<0.001)	−0.28 (<0.001)		
CaPS	-	-		
**IGFBP-3**				
Boyd Orr	0.57 (<0.001)	0.63 (<0.001)	−0.23 (<0.001)	
CaPS	0.39 (<0.001)	0.34 (<0.001)	-	
**Insulin** *				
Boyd Orr	0.06 (0.3)	0.12 (0.021)	−0.44 (<0.001)	0.09 (0.09)
CaPS	−0.02 (0.7)	−0.01 (0.9)	-	0.03 (0.4)

The IGFs and insulin were standardized by: age, sex (Boyd Orr), sample storage time and lab technician (CaPS).

*IGFBP-2 and insulin were natural log transformed before standardization, due to skewed distributions; IGFBP-2 is only available in Boyd Orr.

### Associations of insulin and IGFs with the get-up and go test

All participants in CaPS were male and there was no evidence of a sex-interaction for IGFs with physical performance in Boyd Orr (p-values for the interaction terms ranged from 0.2 for IGF-II to 0.9 for IGF-I); therefore, data were pooled for men and women. In CaPS, a one SD increase in IGF-I was associated with a 2.0% faster get-up and go time after 19 years' follow-up in minimally-adjusted models (95% CI 0.4%, 3.7%; p = 0.017) (Table 3). In the cross-sectional Boyd Orr analysis there was a 1.1% faster get-up and go time per SD increase in IGF-I. However, the confidence interval was consistent with chance (95% CI 1.3% slower, 3.6% faster; p = 0.4). There was no evidence of an interaction between cohorts (Boyd Orr and CaPS) and associations of IGF-I with the get-up and go test, so the data were pooled (p interaction = 0.5). The combined estimate for a SD change in IGF-I was a 1.7% faster get-up and go time (95% CI 0.3%, 3.1% faster; p = 0.017). The coefficient was attenuated to 1.2% faster get-up and go time (0.2% slower, 2.3% faster; p = 0.09) when controlling for confounding variables and to 1.0% faster get-up and go time (95% CI 0.3% slower, 2.4% faster; p = 0.14) after controlling for BMI and co-morbidities (stroke, cancer, diabetes and angina). There was no evidence of a non-linear association between IGF-I and the get-up and go test (p for including a quadratic term = 0.4 in Boyd Orr; p = 0.7 CaPS). There was no evidence of an association between IGF-II and the get-up and go test in either cohort. In Boyd Orr, a one SD increase in IGFBP-2 was associated with 2.6% slower get-up and go times (95% CI 0.4%, 4.8% slower; p = 0.021), but this was only seen when controlling for BMI and co-morbidities. In Boyd Orr, a one SD increase IGFBP-3 in old age was associated with a 3.0% faster get-up and go time (95% CI 0.9%, 5.2% faster; p = 0.007) in fully adjusted models. There was no evidence of any association between IGFBP-3 and get-up and go time in CaPS and there was evidence of a difference between the Boyd Orr and CaPS studies (p interaction = 0.012). A one SD increase in insulin was associated with a 1.3% slower get-up and go time in the confounder-adjusted model (pooled data; 95% CI 0.0%, 2.7%; p = 0.07), but this was attenuated after adjustment for BMI and co-morbidities (coefficient 0.3% faster; 95% CI 1.1% slower, 1.8% faster; p = 0.6). Mutually controlling for IGF-I and insulin did not alter findings (results not shown).

**Table 3:** Associations of age- and sex-standardized IGF z-scores and percentage change in time to complete the get-up and go test from linear regression models.

	Boyd Orr (cross-sectional associations†)			CaPS (prospective associations†)			Boyd Orr and CaPS combined‡		
IGF z-score	%§	95% CI	P	%§	95% CI	P	%§	95% CI	P
**Minimally adjusted models** ¶									
IGF-I	−1.1%	(−3.6, 1.3)	0.4	−2.0%	(−3.7, −0.4)	0.017	−1.7%	(−3.1, −0.3)	0.017
IGF-II	0.1%	(−2.4, 2.6)	0.9	0.3%	(−1.4, 2.0)	0.7	0.2%	(−1.2, 1.7)	0.7
IGFBP-2*	0.4%	(−1.8, 2.7)	0.8	-	-	-	-	-	-
IGFBP-3	−3.1%	(−5.8, −0.6)	0.020	0.5%	(−1.1, 2.3)	0.8	-	-	-
IGF-I∶BP-3	1.6%	(−0.8, 4.11)	0.2	−2.1%	(−3.8, −0.5)	0.031	-	-	-
Insulin*	2.5%	(−0.2, 5.3)	0.068	0.7%	(−0.9, 2.4)	0.3	1.3%	(0.0, 2.7)	0.076
**Confounder adjusted models** ¶									
IGF-I	−0.4%	(−2.9, 1.9)	0.7	−1.5%	(−3.2, 0.2)	0.08	−1.2%	(−2.6, 0.2)	0.09
IGF-II	−0.1%	(−2.4, 2.0)	0.8	0.3%	(−1.3, 2.1)	0.6	0.3%	(−1.1, 1.7)	0.7
IGFBP-2*	0.0%	(−2.1, 2.1)	1.0	-	-	-	-	-	-
IGFBP-3	−2.8%	(−5.1, −0.6)	0.013	0.7%	(−0.9, 2.5)	0.4	-	-	-
IGF-I∶BP-3	2.1%	(−0.2, 4.5)	0.08	−1.7%	(−3.4, −0.1)	0.042	-	-	-
Insulin*	1.3%	(−1.1, 3.8)	0.3	1.1%	(−0.6, 2.8)	0.19	1.3%	(0.0, 2.7)	0.07
**Additionally controlling for BMI and co-morbidities** ¶									
IGF-I	−0.1%	(−2.3, 2.2)	0.9	−1.2%	(−2.9, 0.4)	0.14	−1.0%	(−2.4, 0.3)	0.14
IGF-II	−0.6%	(−2.8, 1.6)	0.5	−0.2%	(−1.8, 1.5)	0.8	−0.2%	(−1.6, 1.2)	0.8
IGFBP-2*	2.6%	(0.4, 4.8)	0.021	-	-	-	-	-	-
IGFBP-3	−3.0%	(−5.2, −0.9)	0.007	0.1%	(−1.5, 1.8)	0.9	-	-	-
IGF-I∶BP-3	2.6%	(0.4, 4.9)	0.020	−1.1%	(−2.8, 0.5)	0.19	-	-	-
Insulin*	−1.4%	(−4.1, 1.3)	0.3	0.3%	(−1.5, 1.7)	0.9	−0.3%	(−1.8, 1.1)	0.6

*IGFBP-2 and insulin were natural log transformed before standardizing due to skewed distributions.

† Sample sizes are based on complete-case analyses for all confounders: Boyd Orr IGFs n = 393, insulin n = 359; CaPS IGFs n = 722, insulin n = 950.

‡ Data were combined if there was no evidence the association differed between the cohorts.

§ The natural log transformation was used on the get-up and go test. Coefficients are interpreted as % change in time per SD change in IGF; a positive % change indicates a slower walk time; a negative % change indicates a faster walk time.

¶ Minimal adjusted models control for age at performance test and sex (Boyd Orr); confounder adjusted models additionally control for research centre, adult social class, diet variables (milk intake, calcium, protein, fat and total energy), exercise, smoking, alcohol consumption; fully adjusted models additionally control for body mass index (BMI) at the time of IGF measurement and co-morbidities at the time of the physical performance tests.

### Associations of insulin and IGFs with the flamingo test

Associations of the insulin-IGF system with the flamingo test are shown in Table 4. The estimated odds suggest a lower risk of poor balance for a one SD increase in IGF-I, but the confidence intervals are consistent with chance (Boyd Orr OR = 0.88 in age- and sex-adjusted model; 95% CI 0.65, 1.20; p = 0.4; CaPS OR = 0.87; 0.73, 1.04; p = 0.13). There was no evidence of an interaction for the associations of IGF-I and cohort for balance ability so the data were pooled (p interaction = 0.9). The combined estimate gave an odds ratio of poor balance of 0.87 (95% CI 0.75, 1.01; p = 0.08). The estimated odds ratio was only slightly attenuated when socioeconomic position, diet, health behaviors, BMI and co-morbidities were controlled for. There was no evidence of associations of IGF-II, IGFBP-2, IGFBP-3 or the IGF-I∶BP-3 molar ratio with balance ability in either cohort, or when data were pooled across cohorts (Table 4). In CaPS, a one SD increase in log transformed insulin was associated with a higher odds of poor balance (OR 1.16; 95% CI 0.99, 1.35; p = 0.059). A similar association for insulin was not observed in Boyd Orr (p interaction = 0.6; giving a pooled estimate of 1.13; 95% CI 0.98, 1.29; p = 0.08 in confounder-adjusted models), which was attenuated when BMI and co-morbidities were controlled for. Mutually controlling for IGF-I and insulin did not alter findings (results not shown).

**Table 4:** Associations of age- and sex-standardized IGF z-scores with the flamingo test from logistic regression models.

	Boyd Orr (cross-sectional associations; n = 392†)			CaPS (prospective associations; n = 725†)			Boyd Orr and CaPS combined‡ (n = 1,117†)		
IGF z-score	OR§	95% CI	P	OR§	95% CI	P	OR§	95% CI	P
**Minimally adjusted models** ¶									
IGF-I	0.88	(0.65, 1.20)	0.4	0.87	(0.73, 1.04)	0.13	0.87	(0.75, 1.01)	0.076
IGF-II	1.05	(0.80, 1.37)	0.7	1.03	(0.87, 1.23)	0.7	1.04	(0.90, 1.20)	0.7
IGFBP-2*	1.08	(0.81, 1.45)	0.6	-	-	-	-	-	-
IGFBP-3	0.94	(0.72, 1.23)	0.7	0.97	(0.82, 1.16)	0.8	0.96	(0.86, 1.11)	0.5
IGF-I∶BP-3	0.88	(0.65, 1.20)	0.4	0.94	(0.79, 1.13)	0.5	0.92	(0.80, 1.17)	0.3
Insulin*	1.06	(0.79, 1.43)	0.7	1.16	(0.99, 1.35)	0.059	1.13	(0.99, 1.19)	0.069
**Confounder adjusted models** ¶									
IGF-I	0.92	(0.66, 1.27)	0.6	0.88	(0.73, 1.06)	0.17	0.89	(0.76, 1.03)	0.10
IGF-II	1.02	(0.76, 1.36)	0.9	1.06	(0.88, 1.27)	0.5	1.04	(0.90, 1.21)	0.6
IGFBP-2*	1.05	(0.79, 1.40)	0.7	-	-	-	-	-	-
IGFBP-3	0.92	(0.70, 1.20)	0.5	0.97	(0.81, 1.17)	0.8	0.96	(0.83, 1.11)	0.6
IGF-I∶BP-3	0.95	(0.69, 1.30)	0.7	0.95	(0.79, 1.15)	0.6	0.94	(0.81, 1.09)	0.4
Insulin*	0.94	(0.70, 1.25)	0.7	1.16	(0.99, 1.35)	0.063	1.13	(0.98, 1.29)	0.084
**Additionally controlling for BMI and co-morbidities** ¶									
IGF-I	0.92	(0.66, 1.30)	0.6	0.92	(0.75, 1.12)	0.4	0.90	(0.77, 1.06)	0.2
IGF-II	1.03	(0.78, 1.37)	0.8	1.04	(0.87, 1.26)	0.7	1.02	(0.87, 1.19)	0.8
IGFBP-2*	1.30	(0.94, 1.80)	0.11	-	-	-	-	-	-
IGFBP-3	0.91	(0.69, 1.21)	0.5	0.92	(0.80, 1.15)	0.4	0.92	(0.79, 1.07)	0.3
IGF-I∶BP-3	0.96	(0.69, 1.33)	0.8	1.02	(0.82, 1.18)	0.9	0.98	(0.84, 1.13)	0.8
Insulin*	0.73	(0.51, 1.05)	0.093	1.08	(0.94, 1.29)	0.4	1.01	(0.87, 1.17)	0.9

*IGFBP-2 and insulin were natural log transformed before standardizing due to skewed distributions.

† Sample sizes are based on complete-case analyses for all confounders: Boyd Orr IGFs n = 392, insulin n = 358; CaPS IGFs n = 725, insulin n = 959.

‡ Data were combined if there was no evidence the association differed between the cohorts.

§ An odds ratio (OR) >1 indicates a relative increase in odds of poor balance; an odds ratio <1 indicates a relative decrease in odds of poor balance.

¶ Minimal adjusted models control for age at performance test and sex (Boyd Orr); confounder adjusted models additionally control for research centre, adult social class, diet variables (milk intake, calcium, protein, fat and total energy), exercise, smoking, alcohol consumption; fully adjusted models additionally control for body mass index (BMI) at the time of IGF measurement and co-morbidities at the time of the physical performance tests.

## Discussion

This is the first study to suggest that higher IGF-I and lower insulin levels measured in mid-life are prospectively associated with improvements in objective assessments of physical performance measured decades later, although the effect estimates were imprecisely estimated (with wide confidence intervals) and so inferences from this study must be made with caution. The associations for insulin were explained by BMI and co-morbidities, though we cannot be certain whether BMI was the upstream determinant of raised insulin or vice versa. There were no associations with IGF-II in either cohort. An association between high IGFBP-2 and slower get-up and go speed was only observed when BMI and co-morbidities were controlled for, so should be interpreted with caution. In the cross-sectional analyses (Boyd Orr), higher levels of IGFBP-3 were associated with faster get-up and go times, but this was not seen in the prospective analyses (CaPS).

The results from CaPS provide some evidence to support the hypothesis that IGF-I is positively associated with levels of physical function in old age, although results from Boyd Orr were consistent with chance. Other studies investigating cross-sectional associations of IGF-I with walking speed have produced conflicting results, with studies showing weak positive effects [5], little association [6], no evidence of an association [7] or a negative association in the oldest-old men [31]. However, IGF-I was strongly positively associated with knee extensor strength in 667 women aged 70–79 years (p = 0.004) [5]. In line with our findings, IGFBP-2 has previously been linked with lower physical performance in a cross-sectional study, which also controlled for BMI [7]. Van den Beld et al. speculated that IGFBP-2 was related to physical performance because it inversely reflected nutritional status (i.e. high IGFBP-2 reflects worse nutritional status) and the biological effects of growth hormone, IGF-I and insulin [7]. In our study an association between IGFBP-2 and the get-up and go test was unmasked when BMI was controlled for, because IGFBP-2 was inversely associated with BMI [12], and in turn, BMI was inversely associated with physical performance [23]. Studies investigating IGFBP-3 with walking speed have not found evidence of an association [6], [32], although IGFBP-3 has been suggested as an indicator of increased catabolism and wasting [33]. The National Health and Nutrition Examination Survey found that higher levels of insulin resistance were associated with slower walking speed in men, in a cross-sectional analysis [34]. Results from the Cardiovascular Health Study showed that higher levels of insulin resistance were associated with markers of frailty (which included an element of slow walking speed) over 9 years' follow-up [35].

IGF-I has a wide range of biological effects on many different tissues, including inhibiting protein degradation in muscle [1]. Therefore, declining IGF-I with age may contribute to loss of muscle strength and reduced physical performance in older adults for tasks that involve power. Results from the Women's Health and Aging Study I and II (cross-sectional data for 728 women aged 70 to 79 years) showed a positive relationship between IGF-I levels and walking speed for IGF-I levels below 50 ng/ml, but only a weak relationship above this threshold [5]. The mechanisms linking insulin with reduced physical performance and frailty are uncertain, but may involve an influence on muscle strength [9].

A main strength of the studies is the objective measures of physical performance, which are known to be associated with risk of falls, progression to disability [36], and death [37] in older people. When there was no evidence of an interaction between studies the data were pooled to increase power. IGFs in CaPS were measured on blood samples taken approximately 19 years prior to the physical performance tests, enabling prospective associations to be examined. However, the blood samples were stored in freezers from the time the samples were taken (from 1984 to 1988) to the assay measurement (in 2009). This long storage time may have had an effect on samples; however, mean levels were in line with IGF levels seen in other samples of men with a similar age distribution [10], [11] and stability studies indicate that IGF levels are stable when stored for 9 years at −80°C [38]. It is likely that any misclassification of IGFs related to storage deterioration would have been random, attenuating observed associations towards the null. A disadvantage in both cohorts is that IGFs were only measured once for each person. However, levels of IGFs track in people over time so a single measurement is reasonably reflective of long-term levels [39]. Blood samples from CaPS phase II were not available for some men with physical performance data. There were no important differences between the men with and without blood samples, in terms of the confounding factors, suggesting that the complete-case analysis is likely to have provided valid inferences. There could, however, be a healthy survivor effect in both cohorts, due to the selective sample of the survivors who were alive at the time of the physical performance tests, probably resulting in conservative estimates between insulin/IGFs and reduced physical performance. The effect sizes observed in this study, and other studies examining IGFs with physical performance, were small. However, even small changes in physical capability can impact on physical dependency, quality of life and medical and social care needs. Times for the flamingo test were dichotomized because no transformation provided an approximate to the normal distribution, but the categorization may have produced a weaker measure of true balance ability and will have reduced statistical power. We did not always observe consistent findings between Boyd Orr and CaPS. This could be for a number of reasons. Firstly, CaPS was prospective with blood samples taken approximately 19 years before the physical performance tests, whereas in Boyd Orr blood samples were taken at the same clinic in which physical performance was measured. The prospective analysis may be considered more robust, as in cross-sectional analyses it is not possible to exclude reverse causation as a potential explanation of associations between IGFs and physical performance. Secondly, the timing of exposure measurement may also have implications, as levels of IGF-I reach a peak in young adulthood and decline continuously with increasing age. The blood samples in Boyd Orr were taken at a mean age of 70.7 years, compared with a mean age of 56.1 years in CaPS and it may be that associations differ by peak IGF levels, mid-life IGF levels or the rate of IGF decline in older age, compared to absolute late-life levels. Thirdly, differences in sample sizes could mean that the smaller Boyd Orr study was underpowered to pick up small effects: confidence intervals in Boyd Orr were, on the whole, consistent with chance. Finally, associations may have arisen by chance due to multiple testing.

If they reflect causal associations and are not due to chance, these findings could have implications for public health interventions: IGF-I has been promoted for treating diabetes, osteoporosis and insulin resistance [40]; however, lifestyle and pharmacological interventions are also being considered to reduce circulating levels of IGF-1 in order to prevent cancer [41]. Potential interventions aimed at raising IGF-I levels in older adults would need to be carefully assessed given the possible adverse effects of raised IGF-I levels on cancer [42]. Increased physical activity has been proposed as a means of influencing IGF-I levels. Weight loss and exercise schemes in people with obesity have been shown to reduce insulin resistance [43]. Such interventions may also result in other benefits such as increasing strength, reducing falls and frailty in older people [4]. Milk intake [44], physical capability and strength [37] have been associated with improved overall survival.

We conclude there was some evidence that high levels of IGF-I, but low levels of insulin, were positively associated with improved physical performance in old age. However, given the large confidence intervals that included the null value, confirmation or rebuttal of the findings in larger prospective studies is needed.
